# Agro-Waste Sweet Pepper Extract-Magnetic Iron Oxide Nanoparticles for Antioxidant Enrichment and Sustainable Nanopackaging

**DOI:** 10.3390/polym16040564

**Published:** 2024-02-19

**Authors:** Elisia María López-Alcántara, Grecia Marcela Colindres-Vásquez, Nouzha Fodil, Marlon Sánchez-Barahona, Octavio Rivera-Flores, Alberto Romero, Johar Amin Ahmed Abdullah

**Affiliations:** 1 Research Management Unit, Agroindustrial Engineering, National Autonomous University of Honduras Technological Danlí, Danlí 13201, Honduras; emlopeza@unah.hn (E.M.L.-A.); greciav942@gmail.com (G.M.C.-V.); marlon.sanchez@unah.edu.hn (M.S.-B.); jorivera@unah.edu.hn (O.R.-F.); 2 Laboratory of Sustainable Management of Natural Ressources in Arid and Semi-Arid Areas, University Center of Salhi Ahmad, P.O. Box 66, Naâma 45000, Algeria; fodil@cuniv-naama.dz; 3 Departamento de Ingeniería Química, Facultad de Física, Universidad de Sevilla, 41012 Sevilla, Spain

**Keywords:** sweet pepper extract, magnetic iron oxide nanoparticles, oxidative stress, banana flour, food preservation

## Abstract

This study synthesizes magnetic iron oxide nanoparticles from agro-waste sweet pepper extract, exploring their potential as antioxidant additives and in food preservation. Iron (III) chloride hexahydrate is the precursor, with sweet pepper extract as both a reducing and capping agent at pH 7.5. Characterization techniques, including microscopy and spectroscopy, analyze the sweet pepper extract-magnetic iron oxide nanoparticles. Antioxidant capacities against 2,2-diphenyl-1-picrylhydrazyl are assessed, incorporating nanoparticles into banana-based bioplastic for grape preservation. Microscopy reveals cubic and quasi-spherical structures, and spectroscopy confirms functional groups, including Fe–O bonds. X-ray diffraction identifies cubic and monoclinic magnetite with a monoclinic hematite presence. Sweet pepper extract exhibits 100% inhibitory activity in 20 min, while sweet pepper extract-magnetic iron oxide nanoparticles show an IC_50_ of 128.1 µg/mL. Furthermore, these nanoparticles, stabilized with banana-based bioplastic, effectively preserve grapes, resulting in a 27.4% lower weight loss rate after 144 h compared to the control group (34.6%). This pioneering study encourages institutional research into the natural antioxidant properties of agro-waste sweet pepper combined with magnetic iron and other metal oxide nanoparticles, offering sustainable solutions for nanopackaging and food preservation. Current research focuses on refining experimental parameters and investigating diverse applications for sweet pepper extract-magnetic iron oxide nanoparticles in varied contexts.

## 1. Introduction

In our quest for environmentally friendly materials with a focus on sustainability, bioplastics emerge as a pivotal solution for addressing the escalating concerns surrounding plastic pollution and non-renewable resource depletion [1,2]. Within this realm, the integration of nanotechnology offers a promising avenue for enhancing the properties of bioplastics and facilitating their efficient decomposition, thereby contributing to a healthier planet [2,3,4,5,6].

Nanotechnology, operating at the nanoscale, involves the study, design, synthesis, and application of materials with unique properties [7,8,9,10]. In the context of plastic waste reduction, nanoparticles play a crucial role, offering diverse possibilities for improving plastic characteristics and expediting their decomposition [11,12,13]. Recognizing this potential, our study explores the synthesis of iron oxide nanoparticles using a green and sustainable approach to contribute to the advancement of eco-friendly materials.

The green synthesis approach, utilizing biological methods such as microorganisms, enzymes, fungi, and plant extracts, aligns with our objective of promoting sustainability [14,15,16,17]. This method, known for its environmentally friendly nature, is employed in our research to obtain metallic nanoparticles, including iron oxide [18,19,20,21,22]. Notably, the green synthesis technique can occur at both intra- and extracellular levels, with plants demonstrating the ability to reduce inorganic ions into metallic nanoparticles on their surfaces and within specific tissues [23,24].

Various methods are employed for preparing nanomaterials, each with its advantages and disadvantages [25]. Chemical methods offer a high control over size and morphology but often involve toxic chemicals and energy-intensive processes [26]. Physical methods provide precise control but are limited by expensive equipment and scalability issues [27]. Mechanical methods are simple and cost-effective but offer limited control over size and shape and consume high energy [28]. In contrast, green synthesis methods stand out for their environmentally friendly nature, utilizing biological entities such as microorganisms and plant extracts. While these methods may have limitations in terms of sensitivity to heating conditions and batch-to-batch variability, their sustainability and biocompatibility make them invaluable for various applications [29].

In the realm of green synthesis, plant extracts offer advantages such as abundance and renewability despite potential challenges related to variability in plant composition and contamination [30]. Microorganisms and enzymes provide specific and controlled synthesis but may face challenges related to slower reaction rates and optimization [31]. The synthesis of magnetic iron oxide nanoparticles through green methods holds particular importance due to their versatile phase, size control, and functional properties [32]. These nanoparticles find applications in drug delivery, hyperthermia treatment, and environmental remediation, showcasing their significance in both biomedical and environmental fields [19,33,34,35]. As nanotechnology advances, the emphasis on green synthesis approaches for nanomaterials, especially magnetic iron oxides, reflects a commitment to sustainable and responsible scientific practices, contributing to the development of innovative and eco-friendly materials [36].

Our focus narrows down to sweet pepper, a vegetable of increasing importance due to its nutritional richness and profitability for producers in agroecological zones. High in vitamin C, along with significant amounts of vitamins A and B and various minerals, sweet pepper becomes a valuable source for our study [37,38]. We leverage the agro-waste derived from sweet pepper, specifically its extract, as a key component in the synthesis of magnetic iron oxide nanoparticles (SPEx-MIONPs). This approach not only addresses the challenge of waste management but also explores the potential of utilizing natural resources for nanomaterial production [39].

As we delve into the synthesis process, our study seeks to demonstrate the feasibility of preparing iron oxide nanoparticles using agro-waste sweet pepper extract. By employing this green method, we aim to showcase the advantages it brings, such as a reduced environmental impact and sustainable nanoparticle production. Additionally, we acknowledge the challenges inherent in this process and emphasize the need for further research to optimize experimental conditions.

Moreover, the utilization of SPEx-MIONPs takes a substantial stride forward as we integrate them into banana-based bioplastics designed for food preservation purposes. The demand for innovative materials like nanopackaging has grown due to environmental concerns with traditional plastics [12]. Beyond environmental benefits, understanding consumer behavior toward nanopackaging is vital for assessing its societal relevance. This amalgamation of agro-waste sweet pepper extract-magnetic iron oxide nanoparticles underscores nanomaterials’ adaptability and aligns with our goal of creating bioplastics from natural sources, particularly bananas [4,40]. As we explore their synthesis and applications, considering consumer perspectives enriches our understanding of the societal context, contributing to the discourse on sustainable materials and their adoption. The demonstrated efficacy of this bioplastic in preserving grapes serves as a tangible illustration of the extensive applications and sustainable advantages that can emanate from our research efforts.

This study unfolds as a comprehensive exploration, weaving together the realms of green synthesis, nanotechnology, and bioplastics. By focusing on the synthesis of iron oxide nanoparticles from agro-waste sweet pepper extract, we aim to contribute to the evolving landscape of sustainable materials, offering a viable and eco-friendly solution for future applications in food preservation and beyond.

## 2. Materials and Methods

### 2.1. Materials

This study employed key components, including 98% iron (III) chloride hexahydrate (FeCl_3_·6H_2_O) from Merck KGaA, Darmstadt, Germany, methanol (CH_3_OH) from Merck Life Science S.L., Madrid, Spain, 99.9% high-purity ethanol from Merck Life Science S.L., Madrid, Spain, sodium hydroxide (NaOH) from PANREAC QUIMICA SAU, Barcelona, Spain, gallic acid (C_7_H_6_O_5_) from Merck Life Science S.L., Madrid, Spain, and 2,2-diphenyl-1-picrylhydrazyl (DPPH) from Merck KGaA, Darmstadt, Germany, all meeting analytical-grade standards.

The Honduran sweet pepper, procured from local markets, is cultivated across various rural regions of Honduras. Characterized by a cream-white color, a flattened and smooth reniform shape, and a diameter ranging from 2.5 to 3.5 mm, the seeds remain centrally attached to the plant. In warm and humid conditions, proper storage is imperative to maintaining the seeds’ germination potency over time [41]. Banana flour was initially obtained following the methodology detailed in [42].

### 2.2. SPEx-MIONPs Synthesis

The SPEx-MIONPs were prepared by mixing 100 mL of a 1 M solution of FeCl_3_·6H_2_O with sweet pepper extract (100 mL). Simultaneously, a 5 M solution of NaOH was added to adjust the pH to 7.5, as needed. The mixture underwent manual stirring and a reaction temperature of 60–80 °C for 3 h until a dark brown precipitate was obtained [43,44]. Following the reaction, the precipitate was separated by filtration, and several washing cycles were performed to collect iron oxide nanoparticles. The nanoparticles were thoroughly dried through a dual process, utilizing an oven at 200 °C for six hours and a muffle at 300 °C for two hours, marking the conclusion of the synthesis procedure. The magnetic iron oxide nanoparticles obtained from the sweet pepper extract have been designated as SPEx-MIONPs.

### 2.3. SPEx-MIONPs Characterization

The characterization of SPEx-MIONPs involved a comprehensive approach utilizing various techniques [43,44,45]. Utilizing a Zeiss EVO scanning electron microscope (Pleasanton, CA, USA), scanning electron microscopy (SEM) was performed to examine the morphology and size of the nanoparticles. For a more detailed analysis of morphology and size, transmission electron microscopy (TEM) was conducted using a Talos S200 microscope (FEI, Hillsboro, OR, USA). Fourier transform infrared spectroscopy (FTIR, Nicolet iS50 FITR Spectrometer, ThermoFisher Scientific, Madison, WI, USA) played a crucial role in obtaining information about the structure of SPEx-MIONPs, enabling the identification of different bioactive functional groups and Fe-O bonds. X-ray diffraction (XRD) patterns, measured on a Bruker D8 Advance A25 diffractometer with a Cu anode (Bruker, sourced from Madrid, Spain), were utilized to confirm the oxide phase and crystallographic structure in the range of 2θ = [15–70°].

This advanced characterization aimed to validate and provide profound insights into the structural and morphological features of SPEx-MIONPs. The combination of SEM, TEM, FTIR, and XRD techniques ensured a comprehensive understanding of the nanoparticles’ properties, contributing to a thorough assessment of their structural integrity and potential applications.

### 2.4. Antioxidant Activity

In the course of the preparation of sweet pepper extract (SPEx) for iron oxide synthesis, we conducted an assessment of its antioxidant activity using the disc diffusion technique against the DPPH free radical [43].

A 0.07 mg/mL concentration of ethanolic DPPH solution was prepared, and 25 mL was spread onto an aluminum plate with a 10 cm diameter. Subsequently, 0.5–1 mL of the SPEx was applied to the center of the plate containing the DPPH solution, causing an immediate color change to clear yellow. The antioxidant activity was then evaluated by measuring the areas displaying a color change, utilizing ImageJ software (v1.53q, NIH, Bethesda, MD, USA).

The assessment method, validated by recording the area, employed the following equation to calculate the inhibition percentage:

(1) $$\;\mathrm{Inhibition}\;\left(\%\right)=\left(\frac{Area_{1}-Area_{2}}{Area_{1}}\right)\times 100$$

here, $Area_{1}$ denotes the overall area of the DPPH solution with a violet color, and $Area_{2}$ indicates the portions that remain unchanged in color over time. A full-color transition to yellow signifies 100% inhibition. This method is of significant value in quantifying the antioxidant capacity of plant extracts, providing essential insights for their potential applications. These applications extend to various areas, such as the production of iron oxide nanoparticles, among other possibilities. In the case of SPEx-MIONPs, the DPPH inhibition was assessed following the protocol outlined in [46], with no modifications.

### 2.5. Preservation of Grapes

Grapes, abundant in nutrients yet susceptible to degradation by microorganisms, were chosen to evaluate the potential of utilizing SPEx-MIONPs secured with banana-based bioplastic as a nanopackaging material for food. To treat the grapes, a solution of banana water (1:3) was prepared and stirred for approximately 10 min at 100 °C. Afterward, the solution underwent filtration using a coffee filter paper, resulting in the collection of the liquid. Subsequently, approximately 0.7% of SPEx-MIONPs relative to the banana flour weight, along with 7 mg of glycerin, were introduced and manually mixed into the banana solution. Finally, the grapes were immersed in the resulting solution for 3 min and left for 144 h at room temperature (28 ± 7 °C and RH = 87%) to carry out the grape shelf-life test.

Every 72 h, photographs were taken, and the weight loss rate (WLR, %) was calculated using the following formula [47]:

(2) $$\mathrm{WLR}\;\left(\%\right)=\left(\frac{W_{Fresh}-W}{W_{Fresh}}\right)\times 100$$

where $W_{Fresh}\;$ represents the weight of fresh grapes, and W denotes their weight after preservation.

### 2.6. Statistical Analysis

Statistical analyses, using GraphPad and IBM SPSS 26 (Released2019. IBM SPPP Statistics for Windows, IBM Corp, Armonk, NY, USA. Version 26.0), indicated significant differences (*p* < 0.05) among observations. One-way ANOVA identified significant differences at a 95% confidence level. Duncan’s statistical analysis, a method applicable in one-way ANOVA, affirmed these significance levels (*p* < 0.05) while also evaluating variance heterogeneity.

## 3. Results and Discussion

### 3.1. SEM

**Figure 1:**
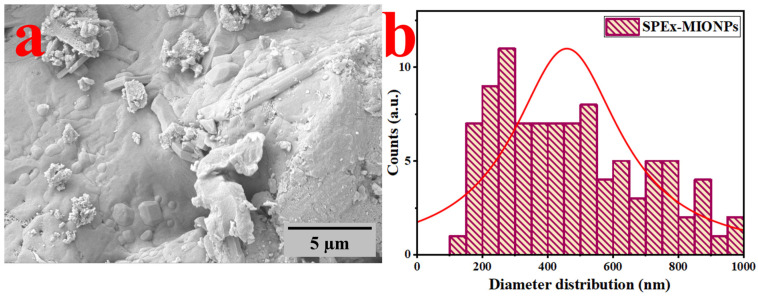
SEM image of SPEx-MIONPs (**a**) and their corresponding diameter distribution (**b**).

In Figure 1, the SEM image of SPEx-MIONPs reveals a range of morphologies, including cubic shapes with some deformation, plate-like structures, flower-like arrangements, elongated rods, quasi-spherical forms, hexagonal structures, and occasional instances of amorphous structures, as shown in Figure 1a.

These distinct morphologies suggest the potential formation of various phases of magnetic iron oxide nanoparticles. Moreover, they indicate the influence of SPEx phytochemical groups, leading to the creation of grains or aggregates during the analysis [48,49]. This alignment is in line with the interaction between the phenolic compound with reducing properties (OH^−^) in SPEx and the surfaces of the different oxide phases of magnetic iron nanoparticles [50]. The surface analysis emphasizes nanoscale irregularities and roughness in the SEM micrographs, offering insights into potential applications such as catalysis or drug delivery [44]. Future studies may benefit from employing methods like atomic force microscopy (AFM) to provide additional quantitative information on surface characteristics [51].

Furthermore, the visual inspection indicates the existence of grains/aggregates, in line with the observed interactions between –OH^−^ in SPEx and nanoparticles [50]. Moreover, SEM images indicate a tendency for the clustering of nanoparticles in specific areas, potentially influenced by electrostatic interactions, van der Waals forces, or solvent evaporation during sample preparation [52]. Approaches to tackle aggregation, like investigating surface functionalization or refining dispersing agents, can be investigated to improve nanoparticle stability and attain a more homogeneous distribution [53].

The grain size distribution, as depicted in Figure 1b, underscores a significant variation in particle size, averaging at 455 nm. The SEM examination offers valuable insights into the structural characteristics and distribution of grain sizes within the synthesized SPEx-MIONPs.

### 3.2. TEM

**Figure 2:**
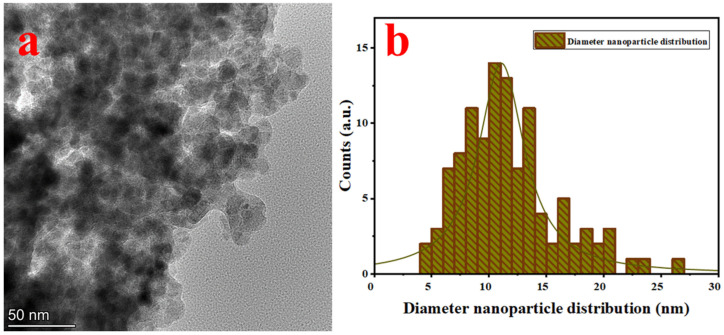
TEM image of SPEx-MIONPs (**a**) and their size distribution (**b**).

The TEM image of SPEx-MIONPs is illustrated in Figure 2, exhibiting diverse morphologies, including quasi-spherical, small cubic, elongated, and hexagonal structures and some with an amorphous morphology (Figure 2a). The nanoparticle diameter distribution histograms, customized with a nonlinear curve fit (Lorentz function), emphasize an excellent dispersion between 4 and 26 nm (Figure 2b).

This notable dispersion is ascribed to the presence of phenolic compounds (–OH^−^) in sweet pepper extract, acting simultaneously as a reducing agent and interacting with nanoparticle surfaces. These interactions significantly enhance iron hydroxide seeds (Fe(OH)), leading to the formation of various magnetic iron oxides, including hematite and magnetite. Consequently, this process mitigates interparticle reactions and the growth rate [50]. The interaction and competition between the phytochemical groups (phenolic compounds) present in sweet pepper extract and iron ions on the surface of their oxide nanoparticles imply an accelerated oxidation rate [54]. The nanoparticle’s diameter distribution, depicted in Figure 2b, indicates an average diameter of 12.5 nm.

### 3.3. FTIR

**Figure 3:**
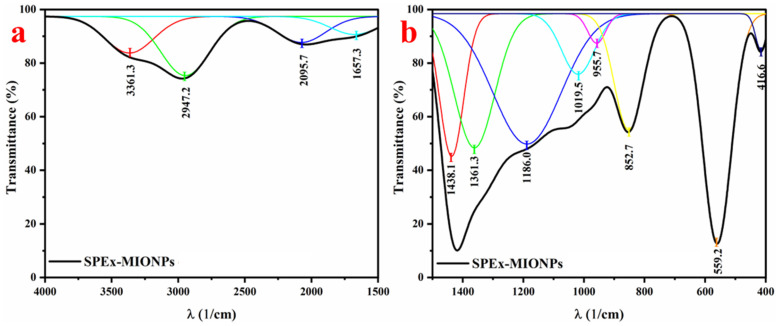
FTIR spectra of the SPEx-MIONPs in the ranges of (**a**) 4000–1500 cm^−1^ and (**b**) 1500–400 cm^−1^. The thickened spectrum in black represents the entire spectra, while peaks in other colors denote the deconvoluted components fitted using Gaussian functions.

The FTIR analysis of the synthesized nanoparticles (SPEx-MIONPs) revealed distinct absorption peaks at various wavenumbers, providing valuable information about the functional groups present in the sample. Fourier self-deconvolution and second (and higher) derivatives were employed, using Gaussian fit functions, to narrow the bandwidths, remove noise, and identify peak centers (Figure 3). The FTIR spectra of the SPEx-MIONPs are depicted in two distinct ranges: 4000–1500 cm^−1^ (Figure 3a) and 1500–400 cm^−1^ (Figure 3b). The absorption band, centered at 3361.3 cm^−1^ and observed in the range of 3500–3000 cm^−1^, signifies the stretching vibrations of hydroxyl (–OH) groups. This broad peak suggests the presence of moisture and polyphenolic molecules adsorbed on the surface of the SPEx-MIONPs [55].

The absorption peak at 2947.2 cm^−1^ is indicative of the symmetric stretching vibrations of methylene (–CH_2_) groups and aliphatic hydrocarbons (CH), commonly present in organic compounds [56]. The absorption bands at 2095.7 cm^−1^ are associated with carbonyl (C=O) stretching vibrations, indicating the presence of ketones, aldehydes, or carboxylic acids [57]. The peak around 1657.3 cm^−1^ may be attributed to C=C [58], the bending vibration of –OH in water [59], and carbonyl (C=O) stretching vibrations [60]. The peak at 1438.1 cm^−1^ suggests the presence of alkene groups (C=C) in aromatic rings, while the peak at 1361.3 cm^−1^ indicates the presence of ester functional groups [50]. Notably, the peaks at 1186.0 and 1019.5 cm^−1^ correspond to C–O–H, C–O, and C–O–C stretching vibrations, indicating the presence of phenolic compounds (–OH) or polysaccharides functional groups [50,61]. The absorption peak observed at 955.7 cm^−1^ may be indicative of the trans-C-H out-of-plane bending vibration [61]. The absorption at 852.7 cm^−1^ may be attributed to aromatic C–H bending [62].

The peaks at 559.2 and 416.7 cm^−1^ suggest the presence of metal oxides, further confirming the synthesis of the mixture phase of magnetic iron oxide nanoparticles (Fe_3_O_4_ and α–Fe_2_O_3_) [56,63,64,65]. These results collectively demonstrate the diverse functional groups present in SPEx-MIONPs, reflecting the complexity of the synthesized material and providing essential insights into its chemical composition and potential applications.

### 3.4. XRD

The X-ray diffraction (XRD) spectra of sweet pepper-based magnetic iron oxide nanoparticles (SPEx-MIONPs) exhibited distinct peaks at 2θ values (18.67, 26.32, 30.13, 31.70, 35.43, 43.01, 53.34, 56.88, 62.68, 65.93, and 68.02°), attributed to the cubic structure of cubic magnetite (space group F d -3 m:2 (227)). These peaks correspond to the crystallographic planes (111), (113), (220), (202), (311), (400), (422), (511), (440), (531), and (442) based on the JCPDS card number 00-901-0939 [66].

Additional peaks at 2θ values of 45.55 and 59.29° were assigned to monoclinic magnetite, corresponding to the crystallographic planes (030) and (049), respectively. The peaks at 21.08, 23.48, 39.33, 40.04, and 54.47° were attributed to monoclinic hematite, with the crystallographic planes (11-1), (20-2), (40-6), (021), and (512), respectively, and the space group C 1 2/c 1 (15) according to the JCPDS card number 00-210-8028 [67].

The calculated particle size using Debye–Scherrer’s equation was determined to be 11.4 ± 3.1 nm. These findings provide detailed insights into the crystalline structure and size of SPEx-MIONPs, essential for understanding their potential applications in various fields.

**Figure 4:**
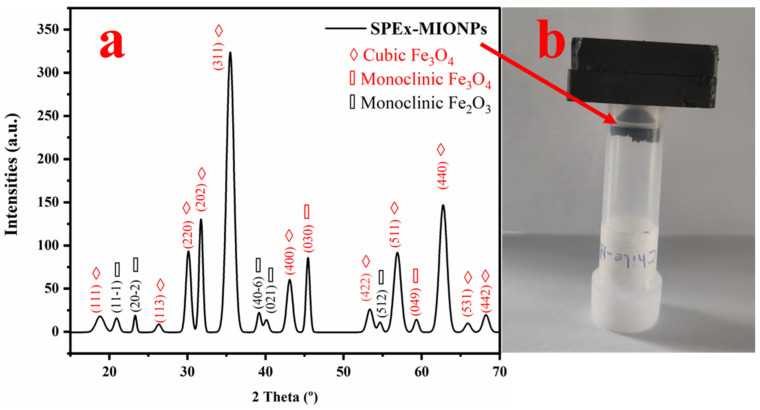
XRD diffractogram of SPEx-MIONPs illustrating distinct crystalline systems and their corresponding crystallographic planes (**a**). The magnetic response of SPEx-MIONPs to the magnet is visually depicted in (**b**).

The XRD analysis of SPEx-MIONPs revealed distinctive peaks at various 2θ values, indicative of the crystalline structure of the magnetic iron oxide nanoparticles. The calculated crystallinity for individual peaks ranged from 0.57% to 32.63%, contributing to an overall total crystallinity of 99.34%. Notably, the prominent peak at 35.43° exhibited the highest crystallinity, contributing significantly to the overall crystalline content of SPEx-MIONPs (Figure 4a). The presence of multiple peaks with varying degrees of crystallinity suggests the formation of diverse crystalline phases within the nanoparticles. These findings provide valuable insights into the structural characteristics of SPEx-MIONPs, paving the way for a comprehensive understanding of their potential applications in various fields. The diverse crystalline phases may influence the nanoparticles’ properties, affecting their performance in catalysis, sensing, and biomedical applications. Further investigation into the specific crystalline phases and their impact on the functional properties of SPEx-MIONPs is warranted for a more nuanced interpretation of their potential applications.

### 3.5. Antioxidant Activity

Table 1 provides a series of photographs capturing the antioxidant activity of sweet pepper extract (SPEx) over time, along with corresponding inhibition percentages against purple DPPH free radicals. The quantitative data, presented at various time points (T_0_ = 0, T_1_ = 1 min, T_5_ = 5 min, T_10_ = 10 min, T_20_ = 20 min), reveals a gradual and effective antioxidant response. Notably, the inhibition percentages demonstrate a substantial increase from 44.1% at T_1_ to 100% at T_20_, indicating a complete color change achieved within 20 min.

The assessment of the antioxidant activity of SPEx using the DPPH free radical assay revealed compelling and time-dependent results. In the first minute, a notable 44.1% inhibition was observed, and this inhibition percentage increased to 52.3 after five minutes, accompanied by a clear color change. At the 10-min mark, the color transition from purple to yellow became more pronounced, signifying approximately 85.8% DPPH scavenging. Despite the negligible standard deviation values over time, the mean inhibition percentages were significant. A complete color change, indicating 100% inhibition, was recorded by the 20th minute, underscoring the potent and time-dependent antioxidant and free radical scavenging properties of SPEx (refer to Table 1).

The observed efficacy of sweet pepper extract (SPEx) can be ascribed to its rich phytochemical composition, encompassing polyphenols, flavonoids, and carotenoids, renowned for their excellent antioxidant properties. The effects observed are likely attributed to the existence of powerful antioxidant compounds [38,68]. The progressive rise in inhibition percentages with time signifies a consistent and evolving antioxidant activity, highlighting the ongoing scavenging of free radicals by the components of the sweet pepper extract. The exceptional antioxidant potential of SPEx is highlighted by the attainment of 100% inhibition at the final time point, signifying the thorough neutralization of DPPH free radicals and affirming its efficacy. A study conducted by Pereira et al. in 2020 [37] utilized a protocol to assess the blocking effect of DPPH in both green and red pepper extracts. In their method, 0.3 mL of the extract with predetermined concentrations of each sample was mixed with 2.7 mL of a methanolic solution containing DPPH radicals, with vigorous stirring at room temperature for 60 min. According to the results of this study, the DPPH radical scavenging activities of red and green conventional peppers ranged from 55% to 71% over the 60-min incubation period.

**Figure 5:**
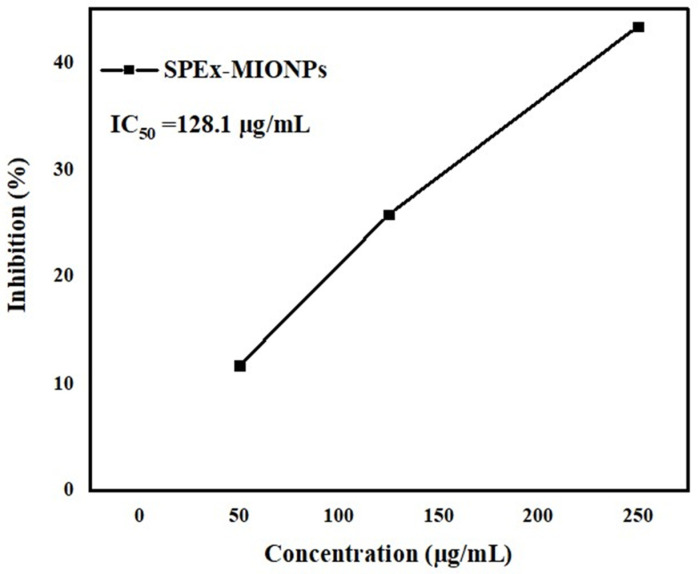
Antioxidant activity of SPEx-MIONPs against DPPH free radicals demonstrated a dependence on concentration.

In comparison, the SPEx obtained in this study demonstrated a continuous increase in inhibition percentages over time, reaching a notable 100% inhibition of DPPH free radicals by the 20th minute. This underscores the robust and prolonged antioxidant effect of SPEx, indicating its potential as a natural source for long-term oxidative stress mitigation. The comparison highlights the unique attributes of SPEx in terms of its sustained scavenging capabilities, showcasing a distinct and potent antioxidant profile. Furthermore, the evaluation of the antioxidant activity of SPEx-MIONPs involved the use of varying concentrations (50–150 µg/mL), as depicted in Figure 5. The nanoparticles exhibited outstanding antioxidant activity, as evidenced by an IC_50_ value of 128.1 µg/mL. This result underscores the concurrent activity of phytochemicals situated on the surface of MIONPs [69]. The observed activity is influenced by various factors, as evidenced by previous methodologies. Notably, the small particle size and a significant proportion of magnetite organized within a cubic crystal system play crucial roles in contributing to the overall effectiveness of the activity. The unique combination of these factors highlights the intricate nature of the system and underscores their collective impact on the observed outcomes [70]. The antioxidant capacity observed also extends its benefits to other properties, including its positive impact on factors like antimicrobial growth, among others [71]. Additionally, a more in-depth analysis of these nanoparticles has been undertaken in conjunction with banana-based bioplastic for the preservation of grapes, as elaborated in the following section.

### 3.6. Grape Preservation

The outcomes of the statistically processed data aim to elucidate the impact of grape preservation using banana-based bioplastic combined with sweet pepper extract-magnetic iron oxide nanoparticles (SPEx-MIONPs). Table 2 illustrates a significant reduction in weight loss rates (WLR) for the SPEx-MIONPs group compared to the control group over the observed time periods. In the initial observation at 72 h, the control group exhibited a weight loss rate of 19.6%, while the SPEx-MIONPs group demonstrated a notably lower rate of 9.7%. This initial difference suggests the potential efficacy of SPEx-MIONPs in mitigating grape mass loss from the outset. As the preservation period extended to 144 h, the control group experienced a significant increase in weight loss, reaching 34.6%, while grape preservation with SPEx-MIONPs maintained a lower weight loss rate of 27.4%. The incorporation of SPEx-MIONPs into banana-based bioplastic enhanced grape preservation by 50.5% at 72 h and 20.8% at 144 h. This outcome highlights the sustained efficacy of SPEx-MIONPs in preserving grapes, with the nanoparticles likely contributing to the antioxidant and preservative properties of the coating.

Comparatively, these results align with those reported by Gvozdenko et al. in 2022 [72], who utilized a gelatin-based nanopacking film with and without CuO NPs. In their study, untreated strawberries exhibited spoilage on the fourth day and tomatoes on the seventh day. Conversely, in samples treated with nanoparticles, there was no spoilage, indicating the inhibition of bacterial and fungal reproduction responsible for spoilage in strawberries and tomatoes. This finding resonates with other studies on nanopacking tomatoes and strawberries using Ag, TiO_2_, and Ti-doped CuO nanoparticles [72]. The collective evidence suggests the potential of various nanopackaging approaches, including SPEx-MIONPs, in extending the shelf life and preserving the freshness of fruits, aligning with the broader advancements in the field.

Despite facing certain limitations related to uncontrollable humidity and other laboratory conditions during the experiment, the utilization of SPEx-MIONPs demonstrated a remarkable preservation capacity. The unforeseen challenges in controlling humidity and maintaining ideal laboratory conditions could have influenced the overall experimental setup. However, even within these constraints, the observed effectiveness of SPEx-MIONPs in grape preservation suggests their robust and significant potential as a preservation agent.

The discussion around these findings underscores the potential of SPEx-MIONPs as an effective solution for improving the shelf life of perishable goods, such as grapes. The antioxidant properties of sweet pepper extract, coupled with the preservative capabilities of magnetic iron oxide nanoparticles, contribute to the observed reduction in weight loss rates. This eco-friendly approach aligns with the growing interest in sustainable food preservation methods, showcasing the promise of SPEx-MIONPs for broader applications in the food industry.

The results from this grape preservation study emphasize the potential of SPEx-MIONPs as a valuable tool for enhancing food preservation and extending the freshness of perishable items. The further exploration and application of this eco-friendly solution in various food industry contexts are warranted, opening avenues for sustainable practices and innovative approaches to food preservation.

## 4. Conclusions

This study successfully synthesized magnetic iron oxide nanoparticles (SPEx-MIONPs) using agro-waste sweet pepper extract as a reducing and capping agent, showcasing their potential as both antioxidants and effective additives in food preservation. The comprehensive characterization through SEM, TEM, FTIR, and XRD elucidated the morphology, structure, and chemical composition of SPEx-MIONPs. SEM showcases diverse morphologies, indicating the potential formation of various magnetic iron oxide nanoparticle phases. Influenced by SPEx phytochemical groups, these shapes suggest grain or aggregate formation. The grain size distribution reveals significant variation, with an average diameter of 455 nm, offering quantitative insights into the structural characteristics of synthesized SPEx-MIONPs. TEM revealed diverse morphologies with excellent dispersion between 4 and 26 nm. The influence of phenolic compounds from sweet pepper extract played a key role, acting as a reducing agent and enhancing iron hydroxide seeds. This interaction resulted in the formation of various magnetic iron oxides, mitigating the growth rate and interparticle reactions. The nanoparticle’s diameter distribution, with an average of 12.5 nm, provides crucial quantitative insights into the structural characteristics of synthesized SPEx-MIONPs. XRD analysis revealed distinct peaks at 2θ values, indicating both the cubic and monoclinic structure of magnetite, as well as monoclinic hematite. The detailed insights into the crystalline structure, along with a calculated particle size of 11.4 nm using Debye–Scherrer’s equation, provide essential information for understanding the potential applications of SPEx-MIONPs.

The antioxidant capacity of SPEx-MIONPs against DPPH demonstrated a notable inhibitory activity, with an IC_50_ value of 128.1 µg/mL. The integration of SPEx-MIONPs into banana-based bioplastic for grape preservation proved to be an innovative and efficient approach. The SEM and TEM analysis revealed the successful stabilization of SPEx-MIONPs within the bioplastic matrix, contributing to the preservation of grapes. The application of SPEx-MIONPs resulted in a significantly lower weight loss rate (27.4%) compared to the control group (34.6%) over a 144-h period, underscoring their potential as a sustainable solution for food preservation and nanopackaging.

This pioneering study not only highlights the natural antioxidant properties of agro-waste sweet pepper but also suggests a promising avenue for the utilization of magnetic iron oxide nanoparticles in tandem with other metal oxide nanoparticles. The encouragement for further institutional research aims to optimize experimental settings and explore diverse applications of SPEx-MIONPs in different scenarios. Overall, the findings of this study contribute to the development of sustainable practices in food preservation and open new possibilities for the integration of agro-waste materials in nanotechnology for broader applications.

## Figures and Tables

**Table 1:** Photographs capturing the antioxidant capacity of sweet pepper extract (SPEx) and the corresponding inhibition percentages against purple DPPH free radicals observed over time. The comprehensive data illustrate the gradual and effective antioxidant response, with a complete color change achieved within 20 min.

T_0_ = 0	T_1_ = 1 min	T_5_ = 5 min	T_10_ = 10 min	T_20_ = 20 min
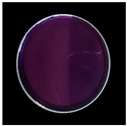	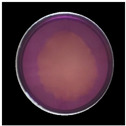	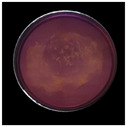	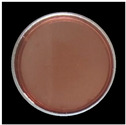	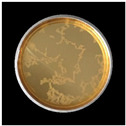
0%	44.1%	52.3%	85.8%	100%

Note: The standard deviation (SD) values remained consistently low over time, indicating negligible variability. Consequently, the statistically significant inhibition percentages were determined based on the mean.

**Table 2:** Visual representation of grape preservation over time, comparing banana-based bioplastic integrated with SPEx-MIONPs and without SPEx-MIONPs (control), along with the corresponding percentage weight loss rates.

Parameters	0 h	72 h	WLR_1_ (%)	144 h	WLR_2_ (%)
Control	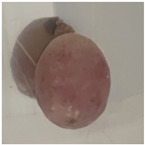	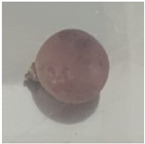	19.6	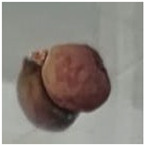	34.6
SPEx-MIONPs	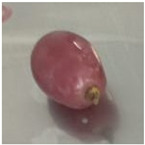	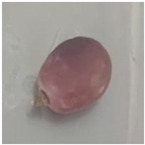	9.7	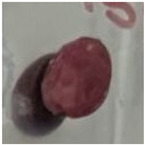	27.4

Note: The standard deviation (SD) values remained consistently low over time, indicating negligible variability. Consequently, the statistically significant weight loss rates (WLRs) were determined based on the mean.

## Data Availability

All data generated or analyzed during this study are included in this published article. Any further specific data analysis can be obtained by making a reasonable request to the corresponding author.
